# Assessing children’s interpretations of the Aboriginal Children’s Health and Well-Being Measure (ACHWM)

**DOI:** 10.1186/s12955-015-0296-3

**Published:** 2015-07-22

**Authors:** Nancy L. Young, Mary Jo Wabano, Stephen D. Ritchie, Tricia A. Burke, Brenda Pangowish, Rita G. Corbiere

**Affiliations:** Laurentian University, 935 Ramsey Lake Road, Sudbury, ON P3E 2C6 Canada; Nahndahweh Tchigehgamig Health Centre, Wikwemikong, ON P0P 2J0 Canada

**Keywords:** Aboriginal, Children, Well-being, Interviews, Questionnaire, Child Self-Report

## Abstract

**Background:**

There are emerging opportunities to improve the health of Aboriginal children and youth. The Aboriginal Children’s Health and Well-being Measure (ACHWM) was developed to enable Aboriginal communities to obtain group-level data from the perspectives of their children 8 to 18 years of age. The survey was developed in collaboration with children, based on the Medicine Wheel framework. The purpose of this study was to ensure that children and youth interpreted the ACHWM questions consistently and accurately and to establish the face validity of the survey.

**Methods:**

Children and parents/caregivers from the Wikwemikong Unceded Indian Reserve (Canada) participated in a detailed interview process as they completed the ACHWM, in 2012. Each participant worked through their thought process verbally, to enable the interviewer to identify questions that were misinterpreted or inconsistently interpreted. Questions were revised based on feedback from the participants, and reviewed with new participants until a stable version was established. The resulting version was reviewed by health care providers and community members to further ensure cultural relevance and face validity within the community.

**Results:**

A total of 18 interviews, with 9 children and 9 caregivers, were required to achieve a stable version of the survey. The children ranged in age from 8 to 18 years. Revisions were required for 19 questions. Most of these revisions were minor linguistic changes. In addition, 6 questions were deleted due to consistent problems and 4 questions were created to address gaps identified during the process. Community members confirmed the appropriateness of the measure for their community and communicated their pride in their youth’s role in the development of this survey.

**Conclusions:**

The result was a 58-question version of the ACHWM that was consistently interpreted and culturally appropriate, and had face validity confirmed by experts from the community, children and their parents/caregivers. The ACHWM is ready to be assessed for relevance to other Aboriginal communities.

## Background

Aboriginal children and youth are the fastest growing demographic group in Canada. Most of the data related to the health outcomes of Canadian Aboriginal children^1^ and youth is aggregated at the national level. When data is examined at the national level significant health inequalities are evident [1–4], despite living in a country that has good health outcomes. Regrettably, Canada’s Aboriginal children face challenges related to the social determinants of health, with higher rates of poverty and lower educational attainment than their age-matched peers in Canada. Aboriginal communities experience higher infant mortality rates, lower rates of immunization, and ever increasing rates of obesity, diabetes and other chronic diseases [5]. How these inequities are expressed at a community level is not clearly understood [6]. This is particularly important since many Aboriginal communities in Canada (particularly First Nations) have begun to control their own health services. These communities also have access to resources and programs to treat illness and promote health. As Aboriginal communities regain control of their health services and programs, they require evaluation tools to inform decision-making.

It is critical that community level data on current health status be obtained. This may be achieved using surveys, provided that they are appropriate for the local culture and context. The resulting data will enable health services directors to identify local needs, tailor programming to meet these needs and to assess the impact of programs and services. Mainstream surveys are not appropriate in the context of Aboriginal communities [3, 7]. Culturally appropriate measures of health, from the perspective of Aboriginal children, are nearly non-existent within Canada and indeed throughout the world [5].

The Aboriginal Children’s Health and Well-being Measure (ACHWM) was developed to address this gap and assess health outcomes from the perspectives of Aboriginal children [8], and to enable children to contribute to health assessment at the community level. The ACHWM was developed as a collaborative research initiative between one Aboriginal community in Canada (the Wikwemikong Unceded Indian Reserve) and an academic institution (Laurentian University), with the intent of being relevant to other communities [8]. Wikwemikong is an Aboriginal community of approximately 7,945 First Nations people, about 40 % of whom live on their traditional lands (www.aandc-aadnc.gc.ca), on Manitoulin Island in northern Ontario, Canada.

The Medicine Wheel [9–12] was identified by the project’s Advisory Committee as the framework for the survey’s development [8], and to ensure that the questions reflect the four quadrants of health: spiritual, emotional, physical and mental. The Advisory Committee was comprised of health leaders in the community, teachers, a local Elder and an academic researcher who was not part of the research team.

The content of the survey was developed through a series of 6 full-day focus groups with children from Wikwemikong, between August and October of 2011 [8]. A total of 38 children were actively engaged in these focus groups via photo-voice activities in which they took pictures that represented each of the quadrants of health. The children spent the afternoon sharing their stories of how the photos represented health. These photos were labeled with key words by the children and placed on a large Medicine Wheel to indicate what quadrant of health they represented. The key words were adapted into questions by the Advisory committee. The final survey was reviewed by a sub-group of 15 children, who were also asked to place the items in quadrants again, to confirm the domain structure. The survey development process also included extensive consultation with community members, Elders and health care workers.

Throughout this process the voices of children and the conceptualization of the Medicine Wheel remained dominant [8]. The final product, the ACHWM, is a self-report survey for children between the ages of 8 and 18 years, that produces quadrant scores and an overall score. The validity of child-self-report has been well established in mainstream populations [13–16].

Child self-report is important to enable children’s voices to contribute to local health assessment and evaluation. Child-self-report is also important to the feasibility of gathering community level data. Thus it is critical to ensure that individual children are able to understand the questions without adult support.

The purpose of this study was to assess children’s understandings of the ACHWM questions and revise as necessary to ensure a clear and consistent interpretation.

## Methods

This study was part of an ongoing program of collaborative research, in the Wikwemikong community. Details on the characteristics of this community have been previously published [17]. This study adhered to principles of community-based participatory research [18–20]. The study was approved by the Laurentian University Research Ethics Board and the Manitoulin Anishinabek Research Review Committee. Written informed consent and assent were obtained for all participants.

The study design included community consultations and detailed individual interviews with children and parents (or caregivers) using a process of cognitive interviewing or debriefing [21, 22]. The cognitive interviewing process has been described in the literature as a valuable method to assess the understandings of children when completing self-report surveys [22]. It was the primary method used in this study, and is described in more detail below. All interviews were completed in the summer of 2012.

### Community consultations

Community guidance was sought repeatedly throughout the process via an Advisory Committee, comprised of community members and healthcare professionals. These members were vital in the development of the survey, and their continued involvement contributed to the cultural relevance of the ACHWM. In addition, we hosted several open community consultation sessions. These sessions enabled community members to provide general feedback, as well as specific suggestions to improve the wording for particular questions.

### Interviews

The purpose of the interviews was to assess whether each of the questions in the ACHWM was consistently and accurately understood, and relevant to the construct that it was intended to measure. Children were recruited from within the Wikwemikong community by a member of the local healthcare team. This was a sample of convenience; however efforts were made to include both girls and boys across the full range of 8 to 18 years. A parent or caregiver for each child was also asked to participate. Both children and parents were required to provide written informed consent. Those with known cognitive impairments, that may have compromised their ability to self-report, were not recruited.

Aboriginal children were invited to participate in a 1-hour cognitive interview with a member of the research team. These interview methods were based on those described by Jobe in 2003 [21], and subsequently applied by other groups [23, 22]. These methods have been successfully applied by members of this group (TAB and NLY) in several cross-cultural studies [24, 25, 22]. A one-day training session was held with all research team members and 3 pilot interviews were conducted to ensure a consistent approach to interviewing, data management and analysis.

At the beginning of the interview, participating children and parents/caregivers were asked to complete a global health rating in which they evaluated their general health as: excellent, very good, good, fair, or poor. This provided a comparison for the results obtained from the survey. After reviewing the results from the first day of interviews, the Pediatric Quality of Life Inventory (PedsQL) [26] was added to the process to provide a second reference score for comparison.

During the interview, each child was probed by the interviewer to “think out loud” and verbalize their thought process related to each question and response as they completed the ACHWM. The children were also asked to give examples to support their answers. This enabled the researchers to assess their comprehension and interpretation of each question. A second member of the team attended the interview and took notes regarding any issues that the participant identified during the interview process.

In addition, we sought to ensure that the parents understood the questions. However, we established *a priori* that if there were discrepant recommendations from the child and parent interviews, the children’s needs would be prioritized. One parent or caregiver of each child also completed an individual interview following a similar format. Each parent (or caregiver) interview was done concurrently with their child’s interview, but in a separate room. These paired interviews were scheduled over 3 days and were held at the Health Centre in Wikwemikong.

#### Coding

During the interview process there were 3 criteria that were used to identify questions for further examination or revision: (1) when a participant struggled to read words out loud; (2) when a participant did not understand words or concepts; or (3) when a participant did not understand the response options. Specific codes were used to identify these observations and were marked on the interviewer’s copy of the survey using a coding system. When a potential problem was identified, the participant was asked to provide suggestions on how to revise and improve the question.

After the interview was complete, these codes were entered into the debriefing database. The database was reviewed in detail after each series of two or three pairs of interviews and revisions were made when a consistent problem was identified by several participants.

Each time a question was revised through this process, a new survey was prepared for subsequent interviews to determine the acceptability of the revisions. All revisions were recorded in the notes section of the database to ensure that every aspect as to why a question was revised was clearly identified. Subsequent revisions were made until a well-understood and stable version of the survey was achieved.

#### Preferred format

Participants were also asked to evaluate various formats of the survey to determine the preferred order and layout of the questions. Three different formats were shown, all of which had slight differences in response set placement, order of questions (organized by quadrant or randomly mixed) and page layout.

### Consensus meetings

At the end of the interviews, a final research team meeting was held to review all of the results. This meeting ensured that all suggested revisions had been documented and addressed appropriately. Question scoring was also reviewed during this time. In addition, Aboriginal experts in the fields of education, mental health, children’s health and community development were invited to review the results. These experts were from the local community and were invited to participate by the Wikwemikong Community Researcher. They ensured key cultural values were retained through the revision process and that the survey remained appropriate from a clinical perspective.

## Results & discussion

Nine pairs of participants (child and parent/caregiver) were interviewed. The children ranged in age from 8.3 to 17.1 years of age with a mean of 12.0 and a standard deviation of 3.2 years. There were 5 girls and 4 boys interviewed. All participants provided written consent and assent. The parents/caregivers included: 6 mothers, 1 father, 1 aunt and 1 grandmother. Most interviews were slightly less than one hour in duration and included rich discussions of the interpretation of all questions. Data from these cognitive debriefing interviews was continuously monitored and reviewed by the research team. Observations were discussed in a research team meeting when consistent problems were evident in the database. This occurred after the 3^rd^, 4^th^, 5^th^, 7^th^ and 9^th^ pairs of interviews.

### What did we learn about the survey’s content?

During cognitive debriefing interviews with 18 participants, 25 questions were identified as being potentially problematic. Revisions had been suggested by many of the participants and were made to 19 questions to improve the interpretation. There were no suggested revisions for 6 questions and these questions were deleted. Most of these revisions (10 of 19 revisions and 5 of 6 deletions) were apparent early in the process, after the 3rd child and caregiver pair had been completed. Most (13) of the revisions and all of the deletions were made as a result of feedback from both children and parents/caregivers, with a few revisions (5) made based on child feedback alone and one revision made due to parent feedback alone. Table 1 shows examples of the types of revisions that were required to achieve a stable and consistently understood version of the ACHWM.

**Table 1:** Examples of ACHWM Queston Revisions

ACHWM question	Problem experienced	Question revision
I feel physically fit…	Word Problem	I feel physically fit (I feel that my body is in good shape)…
My Anishinaabe (Aboriginal) language is…	Word Problem/ Response Set Change	My Native language is…
Knowing a lot about my history and culture is…	Word Problem	Knowing about my culture (like the stories of my ancestors) is…
I drink water to keep me healthy…	Concept Issue/ Response Set Change	Drinking water to keep me healthy is…
I play outside with friends (free play)…	Concept Issue	I am active outdoors…
I feel like giving up - no point in trying…	Concept Issue	deleted
I walk the path of mno-bimaadziwin (the good life)…	Concept Issue	deleted
I have a long-term friendship…	Lack of variability in responses.	deleted
I have access to clean drinking water…	Concept Issue	I can get clean drinking water…

After the first day of interviews, it was apparent that the scores being generated by the survey were lower than what was initially expected. It was discovered that the children who were not overly connected with their culture, scored poorly on the spirituality quadrant, but were otherwise very healthy. The global health rating and the PedsQL scores for these children were good to excellent and prompted the addition of 2 questions within the spirituality quadrant: *“I show respect to the people around me…”* and *“I have time to be with my family…”*. These 2 questions were selected by experts based on suggestions from the ACHWM development research [8], and were added to augment the spirituality section and reflect the importance of core values as part of spirituality.

### What did we learn about the survey’s format?

The participants provided feedback after they completed the survey and both children and parents/caregivers reported that they liked the survey. The questions were deemed to be appropriate based on culture, represented all four quadrants of the Medicine Wheel, and were consistently understood across the intended age range. The format of the ACHWM that was favoured by children was organized by quadrant (spiritual, emotional, physical and mental) and had the response set at the top of each page. Upon careful review, the team recognized that the emotional questions were predominantly negative and thus the children’s preferred format resulted in a large group of negative questions together. The research team, in conjunction with the local Mental Health team in Wikwemikong decided that this was undesirable, and elected to mix the questions. However, because most of the questions shared the same response set, we were able to honour the children’s request to put the response set at the top of each page.

### Consensus meetings

At the end of the interviews we sought input from our Advisory Committee, local health experts and interested community members. It was at this point that the experts recommended that we add 2 new questions to address perceived gaps: “*I feel safe in my community…”* and *“I feel loved by other people around me…”*. These additions were accepted by the research team and brought the final complement of the ACHWM to 58 multiple choice questions.

As a final step, the research team reviewed all of the results. All of the issues raised had been addressed, and we were confident that a stable and well-understood version of the ACHWM had been achieved. Thus, we do not expect detailed interviews to be necessary when implementing the survey in communities with similar cultural heritage. However, we do recommend this as a method to assess cultural relevance in new communities that have significantly different cultural backgrounds (i.e., for Canadian Métis or Inuit children).

#### Automating the survey process

As we prepared to implement the measure on an annual basis, the requirement for manual data entry of survey responses was identified as a challenge to the long-term feasibility of using the survey in Aboriginal communities. The intent of the ACHWM had always been to enable communities to implement the survey and obtain their own data in a feasible and sustainable manner. This was the focus of significant discussion, as were concerns regarding a mechanism to support low literacy. The team agreed that the use of computer technology would provide a mechanism to address feasibility, sustainability and make the process more fun and engaging for children. A tablet application, that works off-line yet communicates with a central server, was developed to implement the ACHWM.

#### Aid to early intervention

Local Aboriginal experts in the fields of education, mental health, children’s health and community development provided critical input and were responsible for an entirely unexpected outcome. The Wikwemikong Mental Health Team identified 17 questions that they believed were important to monitor closely to ensure that children who required further evaluation based on their responses (e.g., children who report always being upset or angry) received appropriate and timely follow-up. Based on this feedback, the research team developed a rapid screening mechanism. This aspect of the survey is intended to be an aid to early intervention and is not intended to be precise enough to enable diagnosis. Local mental health workers agreed to conduct brief assessments to determine if a child requires health care support. These workers provide an essential service that has been integrated into our implementation process to ensure appropriate and timely follow-up within their home community. None of the children who participated in the interviews were identified as needing further assessment. The use of technology to administer the ACHWM also provides a mechanism to quickly screen for key responses on these screening questions using an algorithm developed in consultation with the Mental Health Team.

### Limitations

We recognize that this study was limited to one community and focused on a small group of children and parents. However, this community is large and diverse, as an alliance of Odawa, Ojibway and Pottawatomi nations [17]. Thus, we believe that these participants represented good diversity in terms of age and gender and the results from the second half of the sample confirmed the robustness of the results. It has led to a more detailed understanding of children’s interpretations than is included in typical survey development processes and recommend it be considered by those who are concerned about the relevance of a new measure in a different context.

### Summary of key findings

Overall, 89 % children identified at least one problem. The results from parents/caregivers were similar. In total: 19 questions were revised, 6 questions were deleted due to consistent problems that could not be resolved, and 4 questions were added for a final total of 58 multiple-choice questions. At the completion of the iterative interview and revision process, all problems had been resolved and all questions were consistently understood. When the 58 questions are divided by quadrant of the Medicine Wheel the breakdown is as follows: 15 spiritual, 22 emotional, 12 physical and 9 mental. The 17 screening questions are embedded within the survey.

## Conclusions

The detailed cognitive debriefing process [21, 22] elucidated problems and solutions to ensure the questions were consistently understood. The age group that participated in the interview process (8 to 18 years) allowed for a wide range of perspectives and opinions regarding the modifications necessary for the survey to be consistently interpreted. The interviewing process provided an opportunity to identify and correct wording choices and thus improved the clarity of the concepts and the face validity of the ACHWM. The extensive engagement with children was an essential component of the process.

The detailed consensus meeting and expert consultation processes also allowed for new information and concepts to be brought forward, which may have been overlooked without this step. This led to the addition of 2 new questions being incorporated into the ACHWM based on differing levels of spirituality and connection to culture. These questions added more diversity to the survey, potentially enhancing its applicability to the broader Aboriginal context, including communities on and off-reserve.

The identification of a screening component was an unexpected outcome, but underscores the value of clinical consultation during this rigorous process. The local mental health team will continue to be involved in all future survey implementation events to provide individual assessment and referrals for children who have low scores on those questions. We are grateful for their ongoing support, which it is essential part of the process and critical to respecting the voices of the children. We intend to continue to monitor the number and appropriateness of referrals and make adjustments to optimise sensitivity and specificity.

In addition to these important results, we have also developed an android application that enhances the survey’s appeal to children while eliminating the need for data entry. The data can be quickly uploaded to a secure REDCap server [27] to enhance privacy, while ensuring access to the collaborative research team. The tablet is capable of reading to children who have low literacy levels, and is able to screen for low scores as requested by the Mental Health team. These innovations are the key to sustainability and feasibility over the longer term. The testing of construct validity of the 58-question tablet-based ACHWM has also been completed and results will be forthcoming shortly.

The results presented here establish a stable version of the ACHWM with strong face validity. However, we recognize that there is significant diversity across Aboriginal communities, and therefore recommend that communities interested in implementing the ACHWM, consider utilizing the cognitive debriefing process to ensure the survey is appropriate for their community. We believe that the ACHWM will be a relevant survey to support health related decision-making at the community level.

## Abbreviation

ACHWM: Aboriginal Children’s Health and Well-being Measure

## Footnotes

1 The terms child and children are used in this paper to refer to children and youth between 8 and 18 years of age.

## References

[1] Frohlich KL, Ross N, Richnond C (2006). Health Disparities in Canada Today: Some Evidence and Theoretical Framework. Health Policy.

[2] King M, Smith A, Gracey M (2009). Indigenous health part 2: the underlying causes of the health gap. Lancet.

[3] Loppie Reading C, Wien F (2009). Health Inequalities and Social Determinants of Aboriginal Peoples’ Health.

[4] National Collaborating Centre for Aboriginal Health (2012). State of Knowledge of Aboriginal Health: A review of Aboriginal Public Health in Canada.

[5] Canadian UNICEF Committee (2009). Canadian Supplement to the State of the World’s Children. Aboriginal Children’s Health: Leaving No Child Behind.

[6] Health C (2009). The Importance of Disaggregated Data Prince George.

[7] Smylie J, Anderson M (2006). Understanding the Health of Indigenous Peoples in Canada: Key Methodological and Conceptual Challenges. Can Med Assoc J.

[8] Young NL, Wabano MJ, Burke TA, Ritchie SD, Mishibinijma D, Corbiere RG (2013). A Process for Creating the Aboriginal Children’s Health and Well-Being Measure (ACHWM). Can J Public Health.

[9] Dumont J. First Nations Regional Longitudinal Health Survey (RHS) Cultural Framework, 2005. Ottawa Canada: Assembly of First Nations; 2005. http://fnigc.ca/sites/default/files/ENpdf/RHS_General/developing-a-cultural-framework.pdf

[10] Isaak CA, Marchessault G (2008). Meaning of health: the perspectives of Aboriginal adults and youth in a northern Manitoba First Nations community. Canadian Journal of Diabetes.

[11] Hart MA (1999). Seeking Minopimatasiwin (the good life): An Aboriginal approach to social work practice. Native Social Work Journal.

[12] Hill DL (2006). Sense of Belonging as Connectedness, American Indian Worldview, and Mental Health. Arch Psychiatr Nurs.

[13] Raat H, Mangunkusumo RT, Landgraf JM, Kloek G, Brug J (2007). Feasibility, reliability, and validity of adolescent health status measurement by the Child Health Questionnaire Child Form (CHQ-CF): internet administration compared with the standard paper version. Qual Life Res.

[14] Varni JW, Limbers CA, Burwinkle TM (2007). How Young Can Children Reliably and Validly Self-Report their Health-Related Quality of Life?: An Analysis of 8,591 Children Across Age Subgroups with the PedsQL 4.0 Generic Core Scales. Health & Quality of Life Outcomes.

[15] Young NL, Yoshida KK, Williams JI, Bombardier C, Wright JG (1995). The Role of Children in Reporting their Physical-Disability. Arch Phys Med Rehabil.

[16] Riley AW (2004). Evidence that School-Age Children Can Self-Report on their Health. Ambul Pediatr.

[17] Jacklin K (2009). Diversity Within: Deconstructing Aboriginal Community Health in Wikwemikong Unceded Indian Reserve. Soc Sci Med.

[18] Isreal BA, Schultz AJ, Parker EA, Becker AB, Allen AJ, Guzman R, Minkler M, Wallerstein N (2008). Critical Issues in Developing and Following CBPR Principles. Community-Based Participatory Research for Health: From Process to Outcomes San Francisco.

[19] LaVeaux D, Contextualizing CS, CBPR (2009). Key principles of CBPR meet the Indigenous research context. Pimatisiwin.

[20] Ritchie SD, Wabano MJ, Beardy J, Curran J, Orking A, VanderBurgh D (2013). Community-Based Participatory Research with Indigenous Communities: The Proximity Paradox. Health Place.

[21] Jobe JB (2003). Cognitive Psychology and Self-Reports: Models and Methods. Qual Life Res.

[22] Price VE, Klaassen RJ, Bolton-Maggs PH, Grainger JD, Curtis C, Wakefield C (2009). Measuring Disease-Specific Quality of Life in Rare Populations: A Practical Approach to Cross-Cultural Translation. Health Qual Life Outcomes.

[23] Irwin DE, Varni JW, Yeatts K, DeWalt DA (2009). Cognitive Interviewing Methodology in the Development of a Pediatric Item Bank. A Patient Reported Outcomes Measurement Information System (PROMIS) Study. Health & Quality of Life Outcomes.

[24] Wu R, Zhang J, Luke KH, Wu X, Burke T, Tang L (2012). Cross-Cultural Adaptation of the CHO-KLAT for Boys with Hemophilia in Rural and Urban China. Health Qual Life Outcomes.

[25] Young NL, St-Louis J, Burke TA, Hershon L, Blanchette V (2012). Cross-Cultural Validation of the CHO-KLAT and HAEMO-QoL-A in Canadian French. Hemophilia.

[26] Varni JW, Burwinkle TM, Seid M (2006). The PedsQL 4.0 as a School Population Health Measure: Feasibility, Reliability, and Validity. Qual Life Res.

[27] Harris PA, Taylor R, Thielke R, Payne J, Gonzalez N, Conde JG (2009). Research electronic data capture (REDCap) - A metadata-driven methodology and workflow process for providing translational research informatics support. J Biomed Inform.

